# *Trypanosoma brucei* growth control by TNF in mammalian host is independent of the soluble form of the cytokine

**DOI:** 10.1038/s41598-017-06496-2

**Published:** 2017-07-21

**Authors:** Gilles Vanwalleghem, Yannick Morias, Alain Beschin, David E. Szymkowski, Etienne Pays

**Affiliations:** 1 Laboratory of Molecular Parasitology, IBMM, Université Libre de Bruxelles, Gosselies, Belgium; 2 School of Biomedical Sciences, The University of Queensland, St Lucia, QLD 4072 Australia; 3 Laboratory of Cellular and Molecular Immunology, Vrije Universiteit Brussel (VUB), Brussels, Belgium; 4 Myeloid Cell Immunology Lab, VIB Inflammation Research Center, Gent, Belgium; 5 Xencor Inc., Monrovia, California U.S.A.

## Abstract

Infection of C57Bl/6 mice by pleomorphic African trypanosomes *Trypanosoma brucei and T. congolense* is characterized by parasitemia waves coupled with the production of systemic levels of TNF. This cytokine is known to control *T. brucei* growth, but also to contribute to tissue damage, shortening the survival time of infected mice. Using a dominant-negative version of TNF to discriminate between the effects of the membrane-form versus the soluble form of TNF, we show that the second form is involved in neither parasite control nor induction of liver injury. Therefore, soluble TNF is likely not a major contributor to disease outcome. We propose that membrane-bound TNF is responsible for both *T. brucei* control and host pathology.

## Introduction

Tumor necrosis factor alpha (TNF) is a transmembrane protein that can be released from the myeloid cells surface as a soluble form by a metalloprotease, the tumor necrosis factor-α-converting enzyme (TACE)^1^. Both the membrane-bound (mTNF) and the soluble form of TNF (sTNF) require homotrimerization to be functional^2^.

In different African trypanosome infection models, control of the protozoan parasite burden in mammalian hosts is mediated by interferon-gamma (IFN-γ)^3^, which triggers the classical activation of myeloid cells (M1). Together with other defense molecules such as nitric oxide or reactive oxygen species (NO, ROS), these activated myeloid cells produce TNF as a major effector to control the growth of these extracellular parasites^4–6^. Yet, this inflammatory response can induce severe tissue pathogenicity, with TNF levels being correlated to disease severity in the host. In T. brucei infected TNF−/− mice (lacking both sTNF and mTNF) parasitemia is higher than in wild-type (WT mice), but the immunopathology is reduced^4, 6^. During *T. congolense* infections, the parasitemia is uncontrolled in TNF −/− mice, and survival is drastically reduced^6^.

However, the respective roles of mTNF and sTNF in controlling *T. brucei* and *T. congolense* growth in mice are not yet known.

African trypanosomes can limit the parasite-controlling function of TNF by inducing the production of cyclic AMP (cAMP) in myeloid cells such as macrophages and inflammatory monocyte-derived cells, thereby reducing their capacity to synthesize TNF and restrict *T. brucei* growth^7^. Accordingly, a *T. brucei* cell line rendered defective in cAMP synthesis following transgenic expression of a constitutive dominant-negative adenylate cyclase (ESAG4 DNc) develops parasitemia levels 100 times lower than control (CTRL) parasites. Such attenuation of ESAG4 DNc parasite growth is fully reverted in TNF −/− mice^7^.

During *T. brucei* infection, TNF is posited to control parasite growth in the host by its direct toxicity after uptake into trypanosomes^8, 9^, although this toxic effect could not be reproduced in axenic culture for either *T. brucei* or *T. congolense* ^10^. The activity of recombinant sTNF was not dependent on the TNF receptor binding site of TNF homotrimer, but relied on the TNF lectin-like activity. This activity is mediated by the so-called TNF TIP domain located opposite of the TNF-R binding site of the molecule^8^. Given the lytic effects of recombinant sTNF on *T. brucei*, it was proposed that sTNF is the main effector of trypanosome control^6, 10^.

However, sTNF is not directly toxic to *T. congolense* and is thought to control *T. congolense* growth through induction of nitric oxide^6^. Therefore, the *T. congolense* model is an attractive complement to our investigations using *T. brucei*.

To further address this sTNF hypothesis, we used the biologic XPro1595, a dominant-negative variant of sTNF that is modified at key amino acids involved in the interaction with the TNF receptors TNFR1 and TNFR2^11^. XPro1595 sequesters the native sTNF into inactive heterotrimers and prevents its signaling through TNF receptors, while it preserves the signaling of mTNF, mostly through TNFR2^12^. These properties make it a useful reagent to assess the specific effects of sTNF independent of mTNF^13^. Here, we used this tool to investigate the role of sTNF during the early infection by *T. brucei* or *T. congolense* parasites, since this is the stage where the absence of TNF appears to exhibit the most drastic effects.

## Results

### Injection of XPro1595 inhibits the LPS hypersensitivity effects of infected mice

**Figure 1:**
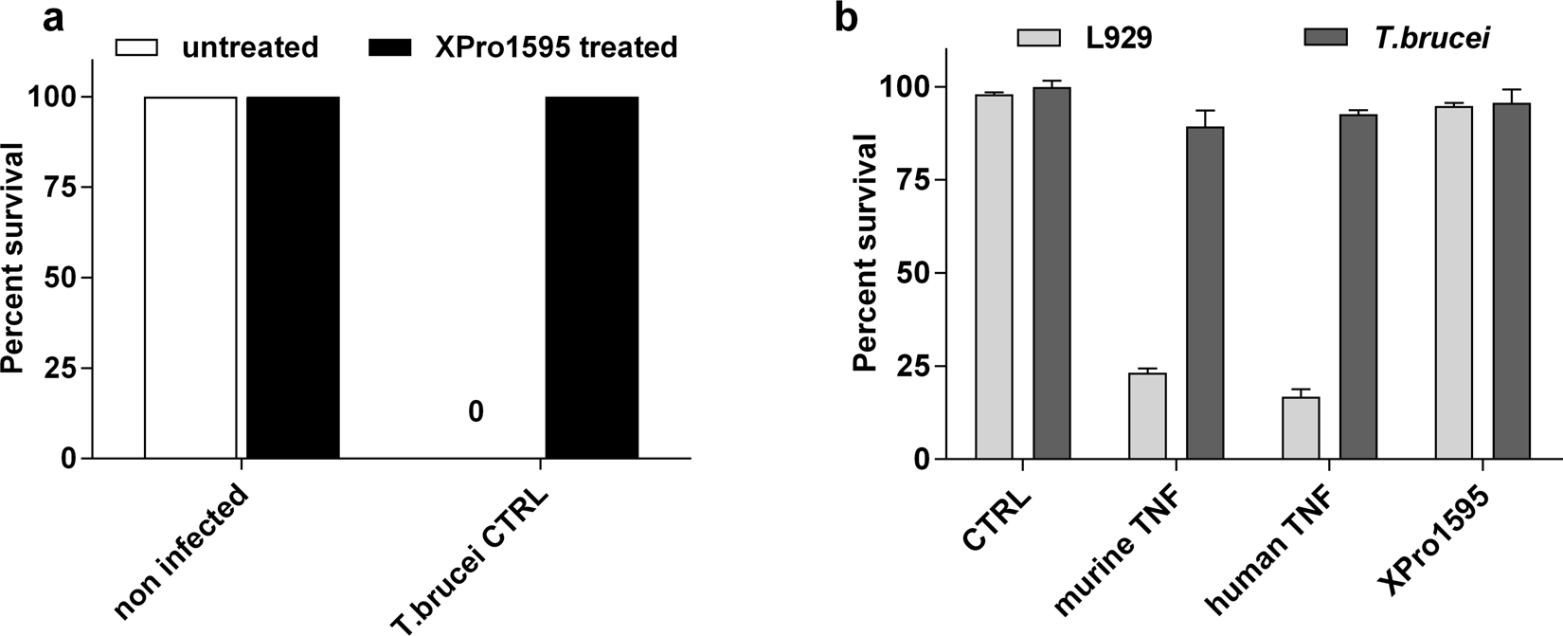
XPro1595 treatment abolishes infected mice hypersensitivity to LPS challenge and does not lyse *T. brucei in vitro*. (**a**) Survival of C57Bl/6 mice infected or not with *T. brucei* control (CTRL) parasites and treated (black) or not (white) with 10 mg/kg of XPro1595. At day 14 of infection, mice were injected ip with 5 µg of LPS and their survival was recorded after 48 h (mean ± SEM, n = 3). **(b)** *In vitro* TNF lysis assay. L929 cells and *T. brucei* parasites were exposed to 50 ng/ml of murine or human TNF, as well as XPro1595 (mean ± SEM, n = 3 for L929 and n = 6 for *T. brucei*). A significant (p-value = 0.0001, Dunnett’s corrected 2-way ANOVA) effect of TNF was observed on the L929 cell line, but no significant trypanolysis was observed.

Due to the induction of TNF expression, *T. brucei* infected mice are a thousand-fold more sensitive to lipopolysaccharide (LPS) challenge than non-infected mice^4^. To validate the efficacy of XPro1595 treatment, which blocks sTNF activity, we tested the sensitivity of C57Bl/6 mice infected with CTRL *T. brucei* with or without treatment with XPro1595. The infected mice that did not receive XPro1595 treatment all died within 48 h of the LPS challenge, whereas all treated infected mice survived (Fig. 1a). This confirms that (i) sTNF plays a major role in the LPS hypersensitivity of *T. brucei* infected mice, (ii) sTNF activity is efficiently blocked *in vivo* by XPro1595, and (iii) a soluble TNF inhibitor protects against a lethal endotoxin challenge^13^.

### XPro1595 does not lyse *T. brucei in vitro*

The trypanolytic effect of sTNF was reported to be mediated by its TIP domain^8^, which is present in XPro1595. As such, we tested the ability of two forms of sTNF to kill *T. brucei* parasites *in vitro*, in comparison with human TNF (hTNF) and murine TNF (muTNF). No effect of either of these TNF molecules was observed on *T. brucei*, whereas both hTNF and muTNF killed L929 cells (Fig. 1b). The lack of direct trypanolytic effect of these recombinant sTNF on *T.brucei* has already been reported^10^, and we show again here no effect of a high concentration of TNF on the parasites in two independent experiments. We also show that XPro1595 has no direct effect on *T. brucei in vitro*.

### XPro1595 treatment has no effect on early parasitemia or early liver injury

**Figure 2:**
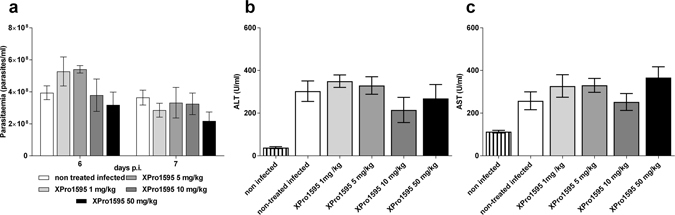
Dose effect of XPro1595 on early parasitemia and liver pathology. (**a**) C57Bl/6 mice were infected with *T. brucei* control (CTRL) parasites and treated with different amounts of XPro1595. Parasitemia was assessed during the first peak at day 6 and 7 pi (mean ± SEM, n = 5), no significant differences were observed with treatment (2-way ANOVA, Dunnett’s correction). **(b**,**c)** ALT or AST serum level at day 7 pi (mean ± SEM, n = 5) by CTRL parasites treated, or not, with different amounts of XPro1595. Non-infected mice serum levels are plotted as a baseline.

A strong increase in parasitemia was observed during the first peak in TNF −/− mice^4, 7^, and this was accompanied by lower morbidity^4^. Various concentrations of XPro1595 were tested for their effect on early parasitemia and early liver injury (Fig. 2). No significant differences were observed on the first peak of parasitemia at the concentrations tested (Fig. 2a). As expected, infected mice showed elevated ALT and AST serum levels (Fig. 2b and c respectively), but XPro1595 treatment failed to prevent early liver injury.

For the rest of our experiments, we chose 10 mg/kg of XPro1595 as the standard treatment since it is the commonly used concentration and could protect mice from the LPS challenge (Fig. 1a).

### XPro1595 treatment in mice does not affect the course of trypanosome infection

**Figure 3:**
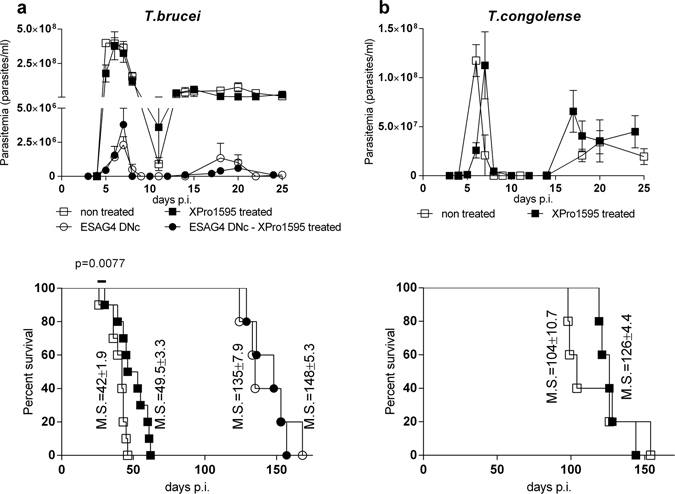
XPro1595 treatment on trypanosome parasitemia and mice survival. Parasitemia (upper panel) and survival time (lower panel) of C57Bl/6 mice either untreated (empty symbol) or treated (black symbol) with 10 mg/kg XPro1595 and infected with: (**a)** *T. brucei* control (CTRL, squares) parasites (mean ± SEM, n = 10), parasites expressing a constitutively expressed DN ESAG4 construct (ESAG4 DNc, circles, mean ± SEM, n = 5), (**b**) *T. congolense* (mean ± SEM, n = 5). A significant, but small (1.179-fold increase, 95% CI 0.4905 to 2.832), increase (p-value = 0.0077 Log-rank test) of survival was observed for the CTRL infected mice after treatment with XPro1595. M.S. = median survival.

C57Bl/6 mice were infected intraperitoneally (ip) with CTRL or ESAG4 DNc *T. brucei* and treated with XPro1595. As illustrated in Fig. 2, *T. brucei* CTRL- and ESAG4 DNc- infected mice developed high and low (100-fold lower) parasitemia, respectively. As reported, host survival increased three-fold in mice infected by ESAG4 DNc parasite compared to the CTRL infected mice^7^. In contrast with the full reversal of the parasitemia and survival phenotype observed in TNF −/− mice^7^, treatment with XPro1595 did not affect the parasitemia in mice infected with CTRL or ESAG4 DNc parasites (Fig. 3a, upper panels). Host survival was significantly increased (1.179-fold increase) in XPro1595 treated mice infected with CTRL parasites (Fig. 3a, lower panel), but not in mice infected with ESAG4 DNc parasites. No significant effect of XPro1595 treatment on parasite burden and mouse survival time was observed during *T. congolense* infection (Fig. 3b).

### Tissue injury is not affected by XPro1595 treatment over the course of the infection

**Figure 4:**
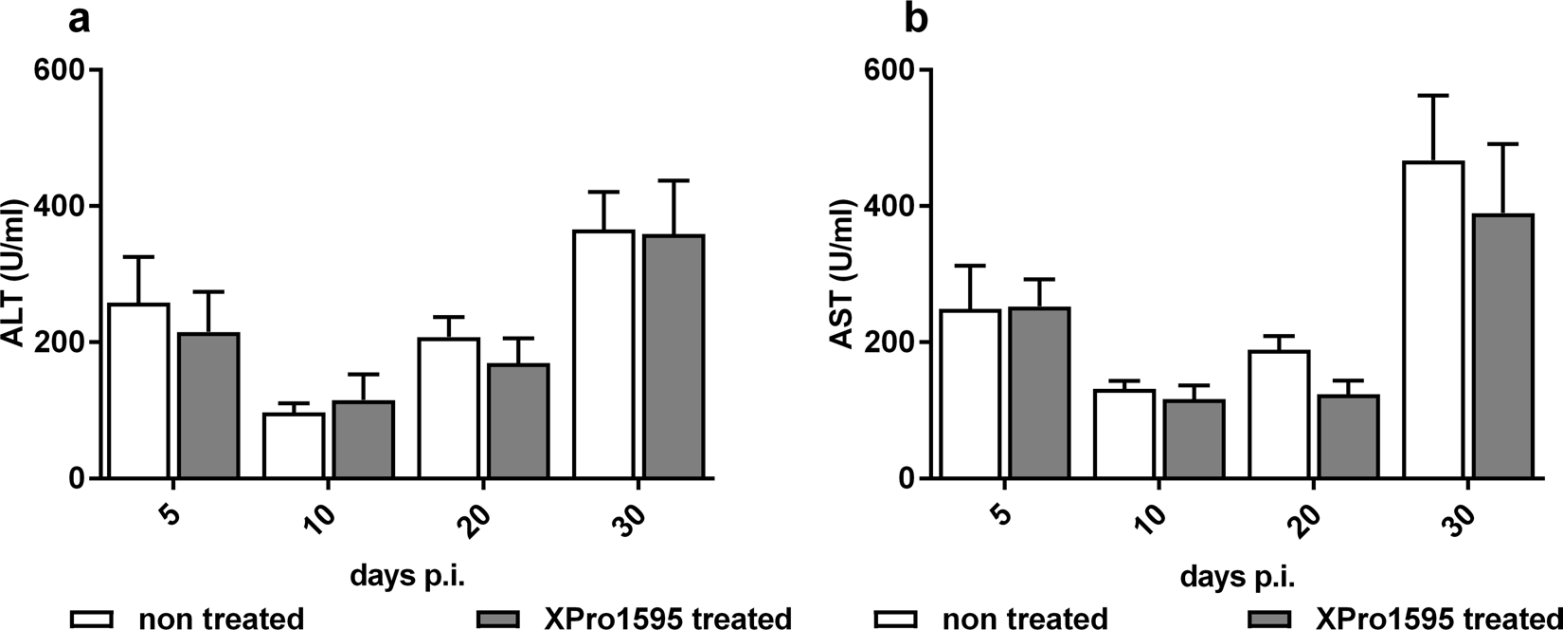
Liver pathology during *T. brucei* infection with XPRO1595 treatment. ALT and AST serum level were measured as a proxy for liver pathology^14^. ALT **(a)** or AST **(b)** serum levels at different time points of a CTRL parasite infection with or without treatment with XPro1595.

As documented^14^, an increase of serum alanine aminotransferase (ALT) and aspartate transaminase (AST) activity, both markers of liver damage with AST also reflecting damage to other organs, was observed at day 7 post-infection in *T. brucei* CTRL-infected mice (Fig. 2b,c). To investigate if lower liver injury would explain the observed increase of survival in CTRL infected mice, we treated mice with XPro1595 and measured the serum ALT and AST levels throughout the infection. Treatment with XPro1595 had no significant effect on the serum levels of ALT or AST of infected mice, although AST showed a trend toward lower late stage levels after treatment (Fig. 4a,b).

### XPro1595 treatment does not affect mononuclear myeloid cell subset subpopulations in the liver in trypanosome-infected mice

**Figure 5:**
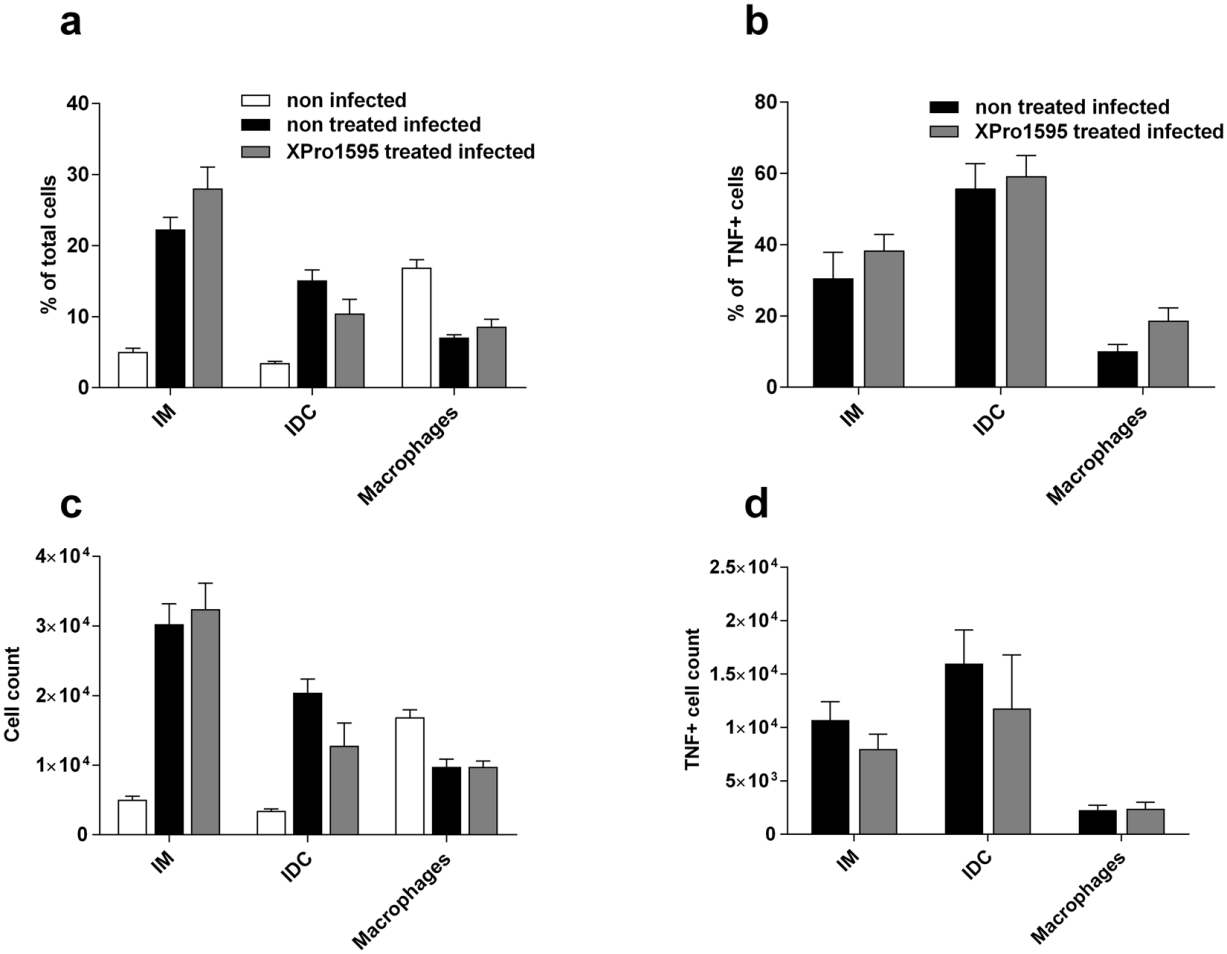
FACS analysis of liver mononuclear myeloid cells subpopulations. FACS analysis of live non-parenchymal liver cells isolated from C57Bl/6 mice at day 5 post *T. brucei* infection after treatment or not with 10 mg/kg XPro1595. Cells were gated and assayed for co-expression of CD11b, CD11c, Ly6C and TNF. **(a)** Percentage of liver CD11b+ mononuclear myeloid cells subpopulations consisting of Ly6C+CD11c− inflammatory monocytes (IM), Ly6C+CD11c+ inflammatory dendritic cells (IDC), and the Ly6C− macrophage; **(b)** Percentage of TNF producing mononuclear myeloid cells in each subpopulation. **(c)** cell count within total CD45+ liver non-parenchymal cells. **(d)** cell count within total CD45+ liver non-parenchymal TNF producing mononuclear cells. All graphs are mean ±SEM, n = 5.

Myeloid cells that secrete TNF contribute to parasite control and to liver pathogenicity during murine African trypanosome infection^7, 15, 16^. Purified liver immune cells from uninfected mice and CTRL *T. brucei*-infected mice were analyzed by FACS following XPro1595 treatment (Fig. 5a). In accordance with previous results^7, 17^, we observed at day 5 post infection an accumulation of Ly6C+ inflammatory monocyte-derived myeloid cells consisting of CD11b + CD11c− inflammatory monocytes (IM) and CD11b+CD11c+ inflammatory dendritic cells (IDC), coupled with a decrease of the macrophage (CD11b+Ly6C−) population. Neither the accumulation of IM or IDC, nor the decrease of macrophage numbers was affected upon treatment of infected mice with XPro1595 (Fig. 5a,c). Moreover, the treatment of infected mice with XPro1595 had no impact on the cell count or percentage of cells producing TNF in IM, IDC or macrophage subpopulations; it is likely sTNF is only inhibited once excreted (Fig. 5b,d).

## Discussion

XPro1595 has been shown to protect *Mycobacterium bovis* Bacillus Calmette-Guérin infected animals from LPS challenge^18^ and the same is shown here to be true for *Trypanosoma brucei* infected animals.

However, contrary to what was observed in other models of infection or inflammation^13, 18, 19^, XPro1595 treatment did not affect the parasite load in *Trypanosoma* infection models. Both acute/trypanosusceptible infection caused by CTRL *T. brucei* and more chronic/trypanotolerant infection caused by *T. brucei* ESAG4 DNc line or by *T. congolense* ^7, 20^ were similarly unaffected by Xpro1595 treatment. These data differ from observation in TNF −/− mice where the early parasitemia increased in mice susceptible to CTRL *T. brucei* infection, as well as in mice more tolerant to ESAG4 DNc *T. brucei* or *T. congolense* infection^6^. These observations call for further studies on why the parasitemia in mice infected with the 3 parasite strains used in this work were affected in TNF−/− mice. In the same vein, the host survival time in chronic/trypanotolerant infection was not affected upon XPro1595 treatment, in contrast to what was observed in TNF −/− mice^6, 7^. However, the survival time was slightly (but significantly) increased in acute/trypanosusceptible infection by CTRL *T. brucei*. The mechanism behind this slight increase in survival is unclear, we speculate it may be through increased TNFR2 signaling, which is not blocked by XPro1595 and can be protective during *T. brucei* infection^11, 12, 21^.

Though ALT levels were not affected upon treatment with XPro1595, we speculate that the slightly decreased AST level in the later stage of infection may reflect lower systemic inflammation of the host. Considering that AST can reflect brain damage, we cannot exclude that blockade of TNF by XPro1595 treatment reduce blood brain barrier damage occurring during *T. brucei* infection, hereby delaying the mortality of trypanosusceptible mice.

Altogether, these data indicate that sTNF does not play a direct role in the control of African trypanosome parasitemia and although AST levels tend to decrease in XPro1595 treated mice, sTNF does not appear to play a major role in early stage infection induced pathogenicity. The absence of an effect of sTNF on parasite growth could have been expected given the presence of high systemic TNF levels during the entire course of infection by *T. brucei* in mice^7, 21^ and the fact that the systemic TNF response could be decreased without affecting parasitemia control during African trypanosome infection^14, 15, 17, 20, 22^. Our data contradict previous *in vitro* studies reporting a direct killing effect of sTNF on *T. brucei* through cytokine uptake by the parasite^8, 9^. Significantly, we used the same methodology and strain of parasite as employed in the previous studies, the only experimental difference appears to be the source of sTNF. These previous studies also proposed that the lectin-like domain of TNF, located opposite of the TNF-R binding site, is involved in *T. brucei* killing. This hypothesis was supported by the observation that *in vivo* treatment of *T. brucei* infected mice with antibodies neutralizing the TNF TIP domain lectin-like activity resulted in increased parasitemia^9^. Although this domain is present in the XPro1595 recombinant protein^11^, no effect on trypanosome growth in mice was observed here following treatment with up to 50 mg/kg XPro1595, which is over the cytokine concentration used in the previous toxicity studies^9^.

As others previously^10^, we also could not reproduce the trypanolytic *in vitro* effect of sTNF on *T.brucei*. As is, it is difficult to speculate why we, and others, failed to reproduce these results. However, if the trypanolytic effect is so sensitive to any change, it raises the question on how broadly it could represent or be significant in an actual physiological response. Moreover, *T. congolense* has been shown to be insensitive to a direct *in vitro* lytic effect of recombinant sTNF^6^. Therefore, we are bound to conclude that the trypanolytic effect of sTNF does not occur *in vivo* nor *in vitro*. Moreover, in a more general context, TNF-dependent control of the early parasite load observed in TNF −/− mice does not likely rely on sTNF. Rather, mTNF could contribute to reduce the *in vivo* fitness of the trypanosome, for example by increased production of reactive nitrogen or oxygen species, facilitating parasite killing by myeloid cells^6, 9, 23, 24^.

Collectively, our data clearly reject the hypothesis of a trypanolytic effect of sTNF both *in vitro* and *in vivo*. It is tempting to speculate instead that the control of early parasitemia may be ascribed to mTNF in both trypanosusceptible and trypanotolerant African trypanosome infection mouse models.

## Material and Methods

Experiments, maintenance and care of C57Bl/6 mice (Janvier) complied with guidelines of the European Convention for the Protection of Vertebrate Animals used for Experimental and Other Scientific Purposes (CETS n°123) and were approved by the Ethical Committee for Animal Experiments of the Université Libre de Bruxelles, Brussels, Belgium (laboratory accreditation number LA2500482).

The mice were inoculated by intraperitoneal (ip) syringe injection of 10^4^ pleomorphic parasites (EATRO 1125 strain, clone AnTat 1.1E, ESAG4 DNc line)^7^, *T. congolense* variant antigenic type 13^25^. Infection parameters (parasitemia, survival) were monitored as previously described^7^.

The dominant-negative TNF (XPro1595) was obtained from Xencor, Inc. It was delivered ip at the specified concentration, or at 10 mg/kg when not specified, one day before the infection and twice a week until day 50 post infection. Injections started one day before the infection as sTNF has been detected at the site of injection one hour after infection^26^. Recombinant murine and human TNF (#315-01 A and #300-01 A, respectively) were from Peprotech (Rocky Hill, NJ, USA).

LPS challenge was assessed as previously described^4^. Briefly, mice were infected intraperitoneally (ip) with CTRL *T. brucei* and treated, or not, with XPro1595 (10 mg/kg) twice per week. After 14 days of infection, mice were injected ip with 5 µg of LPS and their survival was monitored for one week.

Liver mononuclear non-parenchymal cells were isolated and analyzed by FACS as previously described^7, 14^. Alanine aminotransferase (ALT) and aspartate transaminase (AST) levels were measured following the manufacturer’s instructions (Boehringer Mannheim).

L929 mouse fibroblasts cells were cultured in RPMI supplemented by 10% heat-inactivated fetal calf serum, L-glutamine (0.3 μg/ml), streptomycin (0.1 mg/ml), penicillin (100 U/ml) and kept at 37 °C in a 5% CO_2_ humid atmosphere. *T. brucei* parasites for *in vitro* assays were purified from mice and cultured as in^8^. TNF lysis tests were performed as in^8^, using a unique 50 ng/ml TNF concentration. FACS analysis was performed using FlowJo 10. Statistical analyses were performed in Graphpad Prism, sample size is indicated in the figure legends. Results are expressed as mean ± SEM (or median ± SEM for survival). Statistics were assessed by Log-rank test for survival curves, by Kruskall-Wallis one-way analysis of variance followed by Dunn’s post-test or two-way ANOVA followed by Dunnett corrected post-test.

The datasets generated during and/or analysed during the current study are available from the corresponding author on reasonable request.
